# A Poly(ethylenglycol) Functionalized ZIF-8 Membrane Prepared by Coordination-Based Post-Synthetic Strategy for the Enhanced Adsorption of Phenolic Endocrine Disruptors from Water

**DOI:** 10.1038/s41598-017-09364-1

**Published:** 2017-08-21

**Authors:** Mian Wu, Xiafei Guo, Faqiong Zhao, Baizhao Zeng

**Affiliations:** Key Laboratory of Analytical Chemistry for Biology and Medicine (Ministry of Education), College of Chemistry and Molecular Sciences, Wuhan University, Wuhan, 430072 Hubei Province P. R. China

## Abstract

Metal–organic framework (MOF) membranes have received increasing attention as adsorbents, yet single phase MOF membranes have certain limitations, which frustrate their capacity performance. In this work a MOF composite membrane was successfully prepared by a facile and green strategy through reasonable design. At first, a defect-free ZIF-8 membrane was fabricated on an ionic liquid modified pencil bar by a solvothermal method. Then, a novel poly(ethylenglycol) functionalized ZIF-8 composite membrane (ZIF-8/PEG-NH_2_) was prepared through a flexible coordination-based post-synthetic modification strategy. We found that reaction time and temperature were two crucial factors for successfully fabricating well-defined ZIF-8/PEG-NH_2_ membrane. Besides, the adsorption of phenolic endocrine disruptors (e.g., 4-nonylphenol) on original ZIF-8 membrane and ZIF-8/PEG-NH_2_ membrane was investigated, and the good adsorption selectivity of ZIF-8/PEG-NH_2_ membrane towards 4-nonylphenol was demonstrated, with high adsorption capacity and fast adsorption dynamics. Excitingly, such ZIF-8/PEG-NH_2_ membrane was successfully employed for the selective detection of 4-nonylphenol from environmental water samples, demonstrating its great application potential in environmental monitoring.

## Introduction

Metal-organic frameworks (MOFs) are a class of crystalline inorganic-organic hybrid materials with a well-defined porous structure. Compared with conventional inorganic porous materials, MOFs possess higher porosity and specific surface areas^1–3^. Especially, their pore size and surface functionality can be easily tuned upon selection of different metal ions and organic bridging ligands^4–6^. Owing to these unique properties, MOFs have been considered as promising materials for applications in catalysis^7–10^, gas storage/capture^11–16^, separation^17–20^, drug delivery^21, 22^, electronic/opto-electronic devices^23–25^ and sensors^26–29^. In analytical chemistry, the use of MOFs membrane (particularly, ZIF-8 membrane) to selectively remove specific components from mixtures has attracted more and more attention^30–35^.

Among various methods for preparing MOFs membranes, the *in situ* growth with substrate modification has been proven to be a more versatile method for the reproducible preparation of high quality MOF membranes^36–38^. This method can provide sufficient heterogeneous nucleation sites for MOF growth and the formation of continuous membranes. It should be noted that amino terminal compounds (e.g. 3-aminopropyltriethoxysilane)^37, 38^ are widely used as substrate modifiers. For example, Huang *et al*. reported the fabrication of ZIF-90 and ZIF-22 molecular sieve membrane on 3-aminopropyltriethoxysilane-modified support. The obtained membrane exhibited enhanced H_2_ permselectivity. To the best of our knowledge, the use of amino terminal ionic liquid (e.g. 1-(3-aminopropyl)-3-methylimidazolium bromide) as modifiers to prepare a defect-free MOF membrane with high performance remains unexplored. What’s more, in order to improve the binding affinity of MOFs for polar compounds of interest, functional reagents containing poly(ethylenglycol) etc. are necessary to be introduced in preparing more effective materials. However, the preparation of such MOFs encounters multiple challenges by using conventional synthesis methods.

Among the alternative methods for introducing functional groups into MOFs, post-synthetic modification (PSM) approach is of particular interest, and coordinate-based PSM has been employed to produce numerous daughter MOFs that had difficulty to prepare directly^39–41^. Recently, coordinate-based PSM at the SBUs of MOFs has become a mainstream approach for tuning the pore functionality of MOFs. Small amines (e.g. ethylenediamine, abbreviated as EDA) have been applied in the coordinate-based PSM of MIL-101(Cr) and copper (II) triazole MOF^42, 43^. The amine-modified MOF presented enhanced performance and novel properties compared with their pristine counterparts. As small EDA has the ability to access the interior of ZIF-8 crystal, functionalization can be achieved on both the interior and exterior of the material. By contrast, it is difficult for big amines to access the interior of MOF, hence the functionalization can only be achieved on the exterior of the material. In fact, for amino-functionalization linear molecules (such as alpha monomethoxy-omega-amino poly(ethylenglycol), abbreviated as PEG-NH_2_), part of alkyl chain is accessible to the interior of the solids, and the rest part expose on the surface, which lead to the formation of a long superficial PEG “brush”. In addition, PEG-NH_2_ is a very important functional material with hydrophilic properties, especially for applications in adsorption^44–46^. Predictably, such MOF/PEG-NH_2_ heterostructures should possess potential application in adsorption with a high selectivity toward molecules of different sizes.

**Figure 1:**
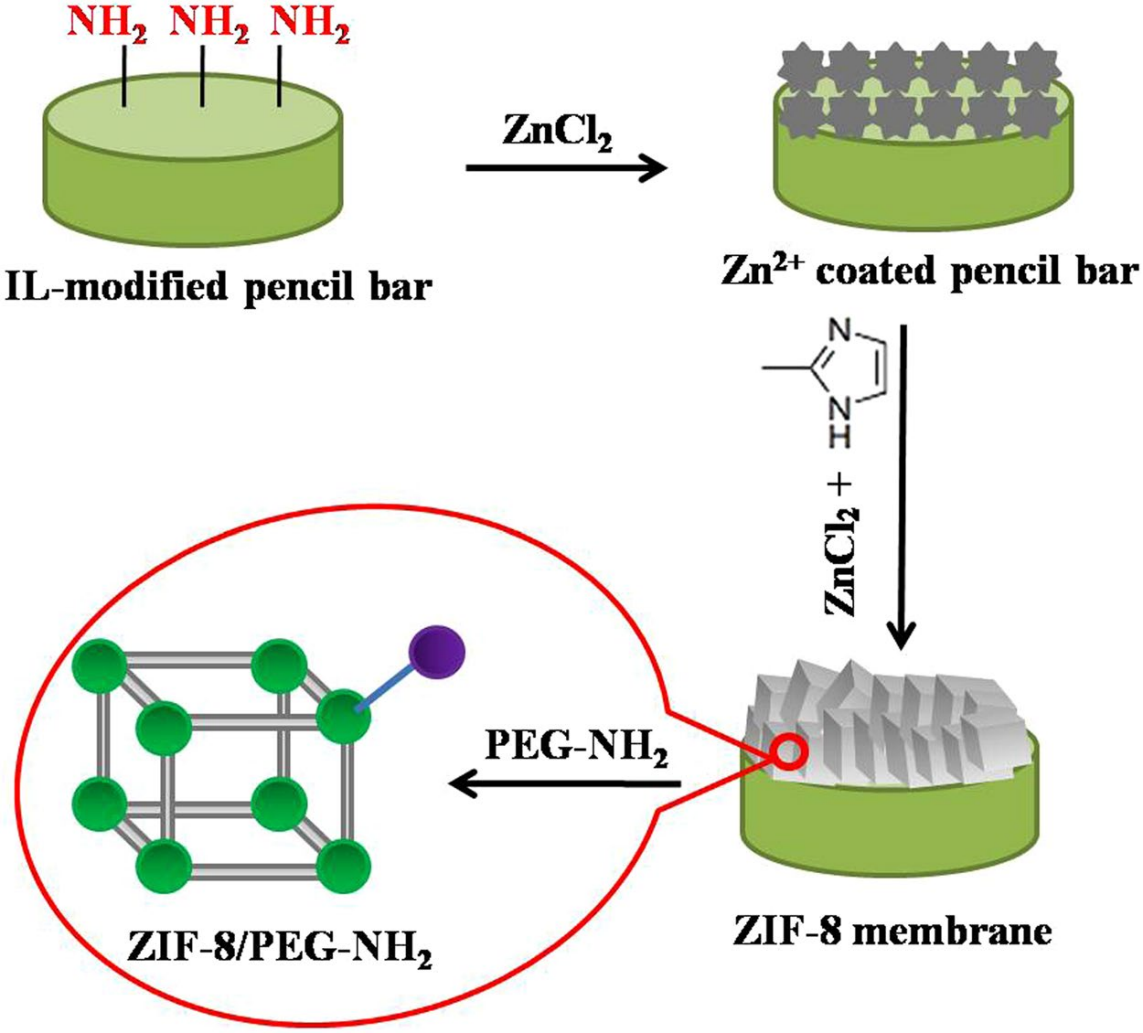
Schematic diagram of the preparation of PEG-NH_2_-functionalized ZIF-8 membrane.

In this paper, we first fabricated ZIF-8 membrane on ionic liquid (IL)-modified pencil bars through *in situ* solvothermal growth. Then we reported a facile coordinate-based PSM strategy for the functionalization of ZIF-8 membrane with PEG-NH_2_, which cannot be obtained easily through direct synthesis (Figure 1). During the process, the reaction time and temperature was fully optimized. Excitingly, such ZIF-8/PEG-NH_2_ membrane exhibited enhanced adsorption properties for phenolic endocrine disruptors (using 4-nonylphenol as model) compared with original ZIF-8 membrane. Accordingly, it was successfully applied to the detection of 4-nonylphenol in environmental water samples. In addition, the ZIF-8/PEG-NH_2_ membrane possessed good stability and reusability. Therefore, it is a promising material for application in environmental monitoring.

## Results and Discussion

### Preparation of ZIF-8 membrane

**Figure 2:**
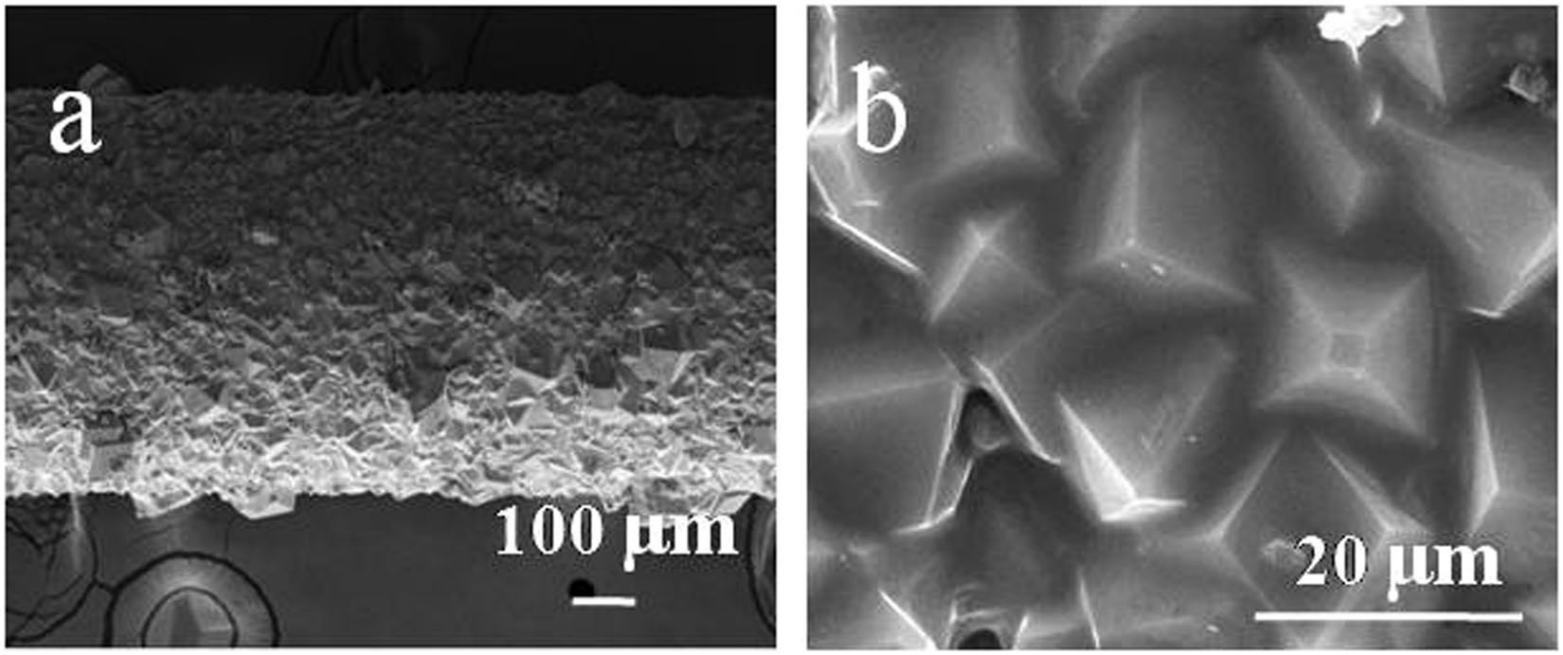
SEM images of ZIF-8 membrane grown on IL-modified pencil bar. (**a**) low magnification, (**b**) high magnification.

By simple immersion in IL solution at room temperature for 0.5 h, the surface of pencil bar was completely covered by a uniform IL layer (Supplementary Figure S1a and b). Then a ZIF-8 membrane was prepared on the IL-modified pencil bar through a solvothermal method. As shown in Fig. 2a and b, the surface of IL-modified pencil bar was covered with well intergrown rhombic crystals, and no visible cracks, pinholes or other defects were observed. Similarly, we attempted to grow a continuous ZIF-8 layer on IL-free pencil bar, but failed. The ZIF-8 crystals were uncontinuous (Supplementary Figure S1c and d) and they stacked together. This indicated it was easier for ZIF-8 crystals to grow on IL-modified pencil bar than on bare pencil bar. In this case, the amino-groups of IL could coordinate to the free Zn^2+^ centers and bound to the growing nano-crystals directly. Therefore, covalent bonds (Zn–N) between the growing ZIF-8 layer and the support were introduced to anchor the ZIF-8 crystals for membrane formation^38^.

### Preparation of ZIF-8/PEG-NH_2_ membrane

**Figure 3:**
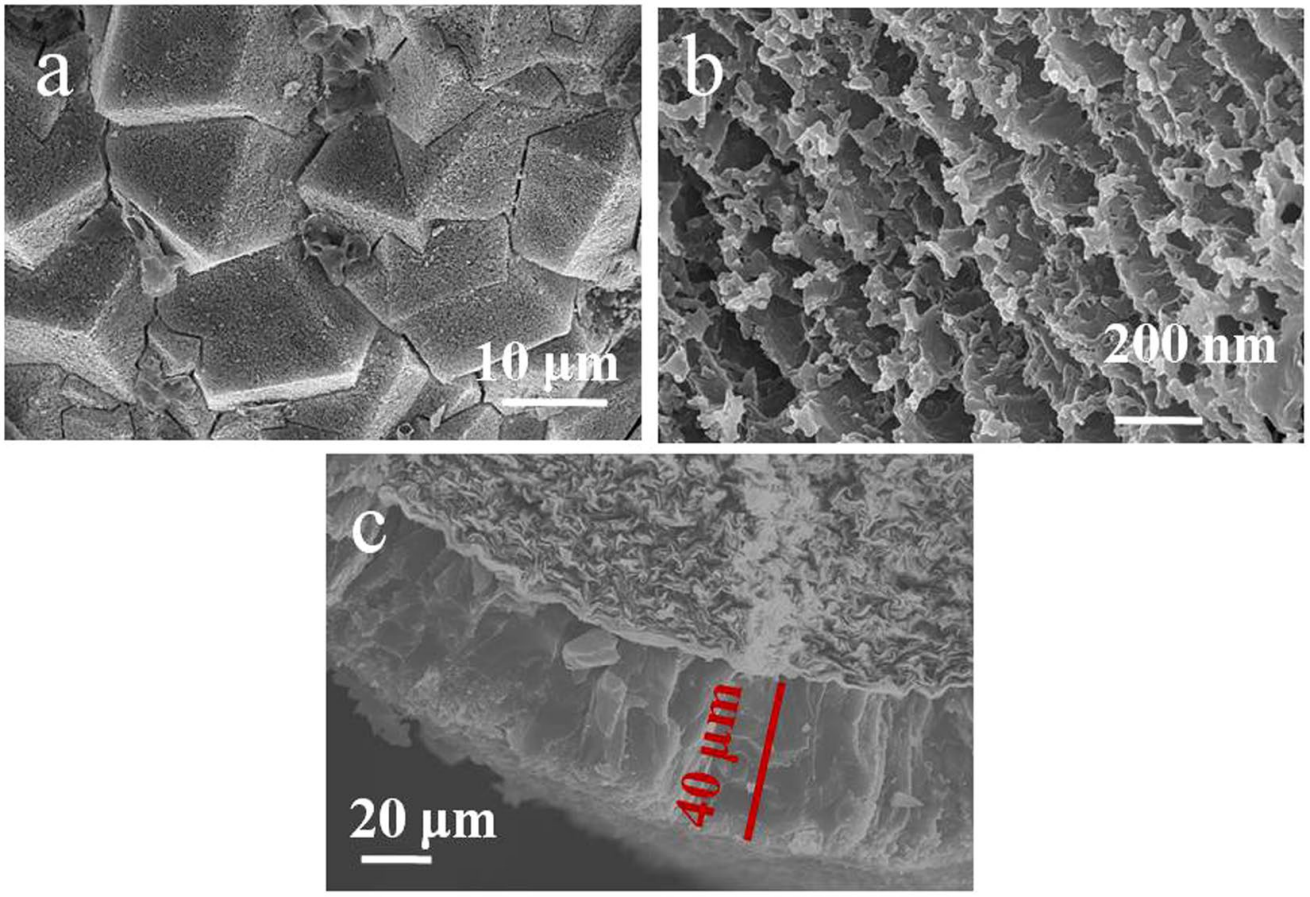
SEM images of ZIF-8/PEG-NH_2_ membrane. (**a**) low magnification, (**b**) high magnification, (**c**) cross section.

**Figure 4:**
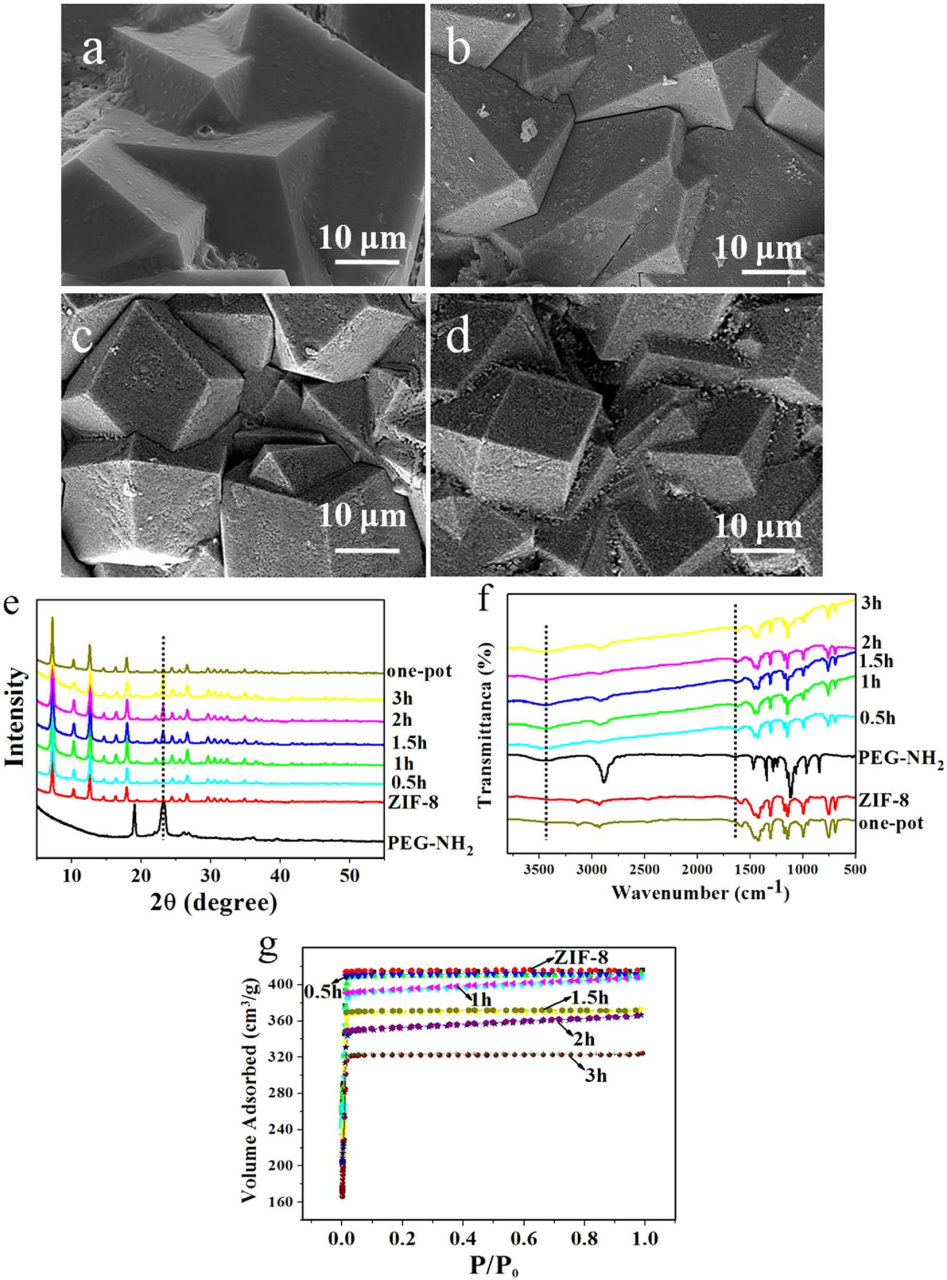
SEM images (**a**–**d**) of the prepared ZIF-8/PEG-NH_2_ composites. Reaction time: (**a**) 0.5 h, (**b**) 1 h, (**c**) 1.5 h, (**d**) 2 h. XRD patterns (**e**), FT-IR spectra (**f**) and N_2_ adsorption–desorption isotherms (**g**) of the prepared ZIF-8/PEG-NH_2_ composites.

After dipped in PEG-NH_2_ methanol solution at 60 °C for 3 h, holes evenly dispersed on the surface of ZIF-8 membrane (Figure 3a). Closer examination (Figure 3b) revealed that fragments were aligned with gaps of ca. 100 nm. It was noticeable from the cross-section view (Figure 3c) that the crystals were tightly anchored to the substrate with a thickness of ∼40 μm, indicating a strong adhesion to the pencil bar. The formation of a pure and highly crystalline ZIF-8/PEG-NH_2_ membrane was confirmed by X-ray diffraction (XRD), which indicated that all peaks match well with those of ZIF-8 and PEG-NH_2_ (Figure 4e). The formation of ZIF-8/PEG-NH_2_ membrane was also confirmed by FT-IR spectra (the peaks of 1600 and 3400 cm^−1^ corresponding to (-NH-), or (-NH_2_) originated from PEG-NH_2_) (Figure 4f). For the introduction of PEG-NH_2_ into ZIF-8, we tried to prepare ZIF-8/PEG-NH_2_ by a one-pot strategy and modulated synthesis first. But the characteristic peaks of the obtained product in XRD patterns and FT-IR spectra (Figure 4e and f) were similar to those of ZIF-8, no peaks of PEG-NH_2_ were found, suggesting that ZIF-8/PEG-NH_2_ couldn’t be obtained easily through direct synthesis.

### Influences of the reaction conditions on ZIF-8/PEG-NH_2_ membrane

Herein we took the reaction temperature of 60 °C as an example to explore the effect of reaction time on the morphology of ZIF-8/PEG-NH_2_ membrane. The results were shown in Fig. 4. After reacting for 30 min, the surfaces of the crystals were almost smooth as the pristine ZIF-8 crystals (Figure 4a). However, after 1 h, several small holes formed on the crystal surface (Figure 4b). With the increase of reaction time, more PEG-NH_2_ molecules diffused to the crystal surface, the number and size of the holes (which were formed by the accumulation of PEG-NH_2_ molecules) increased and the holes expanded deeply into the crystals (Figure 4c–d). However, the contact sites on the surface were limited, after 3 h, it reached saturation, and a great number of holes were generated. Afterwards, the morphology was maintained and no longer changed. Therefore, 3 h was selected.

Furthermore, the intensity of PEG-NH_2_ peak in the XRD patterns increased with extending reaction time (Figure 4e). The FT-IR spectra (Figure 4f) of these ZIF-8/PEG-NH_2_ composites were similar. N_2_ adsorption–desorption was also performed to investigate the pore structure parameters of the as-prepared ZIF-8/PEG-NH_2_ membrane and the results were presented in Figure 4g and Table 1. The surface area and pore volume reduced with the introduction of PEG-NH_2_, this was due to partial filling (or blocking) of the pores of ZIF-8. When the reaction time increased, more pores of ZIF-8 were filled/ or blocked, so the surface area (including interior surface of pores) and pore volume reduced gradually. The elemental analysis showed that the nitrogen content (wt. %) of the composites increased with increasing reaction time, and all of them were larger than that of virgin ZIF-8 (Table 1).

**Table 1:** Surface area, pore volume and nitrogen content of the virgin ZIF-8 and ZIF-8/PEG-NH_2_ composites.

Material	BET surface area(m^2^/g)	Pore volume (mL/g)	Nitrogen content (wt. %)
ZIF-8	2097	1.43	8.65
ZIF-8/PEG-NH_2_ (0.5 h)	1916	1.21	9.86
ZIF-8/PEG-NH_2_ (1 h)	1730	1.06	10.31
ZIF-8/PEG-NH_2_ (1.5 h)	1402	0.96	11.04
ZIF-8/PEG-NH_2_ (2 h)	1243	0.83	12.66
ZIF-8/PEG-NH_2_ (3 h)	1195	0.77	13.75

The molecular size of PEG-NH_2_ is larger than the pore aperture of ZIF-8 (3.4 Å), therefore, it is difficult for PEG-NH_2_ to diffuse or transfer deeply into the inner crystal cavities. Only part of alkyl chain is accessible to the interior of the solids, the rest part exposes on the surface, which leads to the formation of a superficial PEG “brush”. By contrast, small EDA has the ability to access the interior of ZIF-8 crystal, thus functionalization can be achieved on both the interior and exterior of the material. It was noticeable from the SEM view (Supplementary Figure S2a and b) that holes (50 ± 10 nm. O.D.) uniformly dispersed on the surface of ZIF-8 membrane. XRD patterns of ZIF-8/EDA (Supplementary Figure S2c) revealed similar results to those of ZIF-8, indicating that the structure of parent ZIF-8 was well retained after the functionalization process. Compared with ZIF-8, the as-prepared ZIF-8/EDA showed a reduced BET surface area (714 m^2^/g), probably due to the partial blocking of the pores by flexible amine group pendants (Supplementary Figure S2d).

**Figure 5:**
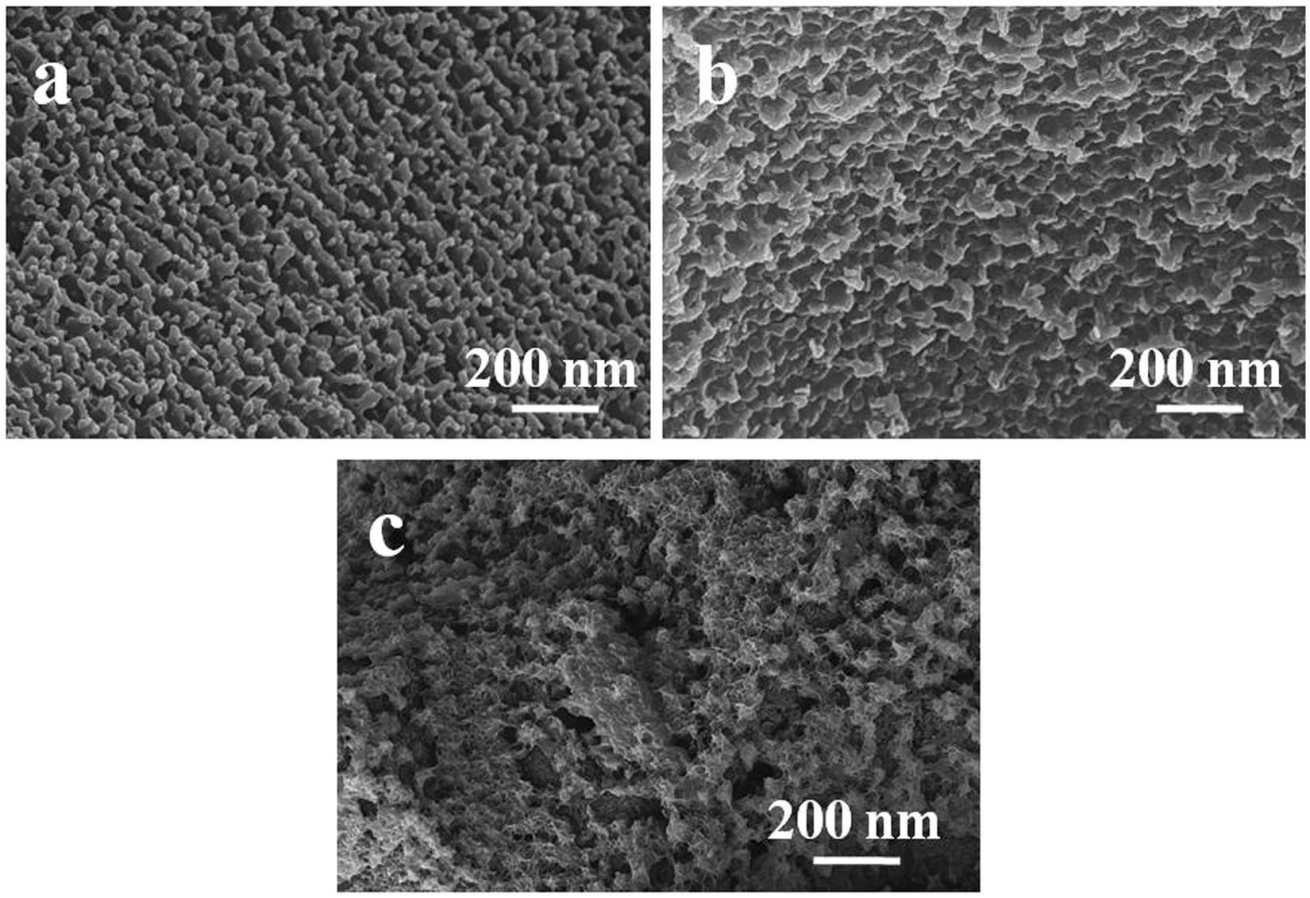
SEM images (**a**–**c**) of the ZIF-8/PEG-NH_2_ composites prepared at different temperatures. Temperature: (**a**) 20 °C, (**b**) 40 °C, (**c**) 80 °C.

Reaction temperature also influenced the morphology of ZIF-8/PEG-NH_2_ membranes by influencing diffusion rate and complex rate. At 20 °C, the spherical particles had a homogeneous arrangement, with each particle having a diameter of ca. 20 nm (Figure 5a). After the temperature increased to 40 °C, these particles became larger with diameter of about 40 nm (Figure 5b). The reason was that, at higher temperature, diffusion rate and complex rate were accelerated, more PEG-NH_2_ molecules were combined by the surface of ZIF-8, large particles were formed due to the combination of small microparticles. At 60 °C, neatly arranged fragments were formed. However, when the temperature was further increased to 80 °C, the reaction was so tempestuous that the fragments arranged in a random way (Figure 5c).

To study the role of PEG-NH_2_ in the process, the pristine ZIF-8 membrane was treated in methanol without PEG-NH_2_ (described as ZIF-8/CH_3_OH) at 60 °C for 3 h. As can be seen in the SEM image and XRD pattern, almost no change for the ZIF-8 crystals occurred (Supplementary Figure S3a and c, respectively). Besides, the N_2_ adsorption-desorption experiment revealed that the surface area of the obtained composite was very close to that of pure ZIF-8 crystal (2033 m^2^/g). It is therefore clear that PEG-NH_2_ plays a critical role in the formation of hole structures.

Moreover, bound PEG-NH_2_ could be removed only after crystals degradation under acidic conditions, supporting the fact that it was firmly bound to the crystals through the coordination of its amino end-group with the metal centers. Indeed, when mPEG was added to the reaction mixture, a negligible surface modification occurred (Supplementary Figure S3b). The XRD patterns in Supplementary Figure S3c only presented the characteristic peak of ZIF-8 crystal, which was in agreement with the SEM image. Besides, the N_2_ adsorption-desorption experiment revealed that the prepared ZIF-8/mPEG composite had a surface area of 2089 m^2^/g, which was almost the same as that of pure ZIF-8 crystal (Supplementary Figure S3d).

### Thermal stability measurements

**Figure 6:**
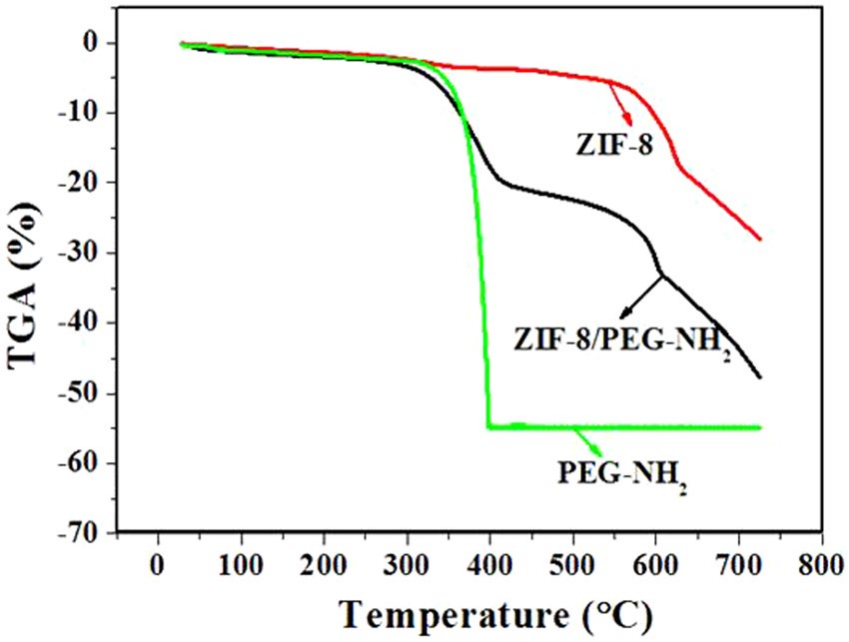
TG curves of PEG-NH_2_, ZIF-8 and ZIF-8/PEG-NH_2_.

The thermal stability of the ZIF-8/PEG-NH_2_ membrane was evaluated by TGA, and the TG curves of the prepared composites are shown in Fig. 6. As can be seen, the weight of PEG-NH_2_ keeps almost unchanged when the temperature increases from 100 °C to 300 °C, and then it declines sharply, corresponding to the decomposition of the skeleton in PEG-NH_2_. ZIF-8 is stable up to 420 °C, which is consistent with that reported in the literature^47^. The ZIF-8/PEG-NH_2_ exhibits two steps of weight loss. The first one occurs at 300 °C, resulting from PEG-NH_2_ decomposition. The another step of weight loss is assigned to the decomposition of ZIF-8, which is similar to that of pure ZIF-8. The results indicate the successful modification of PEG-NH_2_ on the surface of ZIF-8 and the prepared ZIF-8/PEG-NH_2_ membrane exhibits a good thermal stability.

### Adsorption properties of ZIF-8/PEG-NH_2_ membrane for phenolic endocrine disruptors

**Figure 7:**
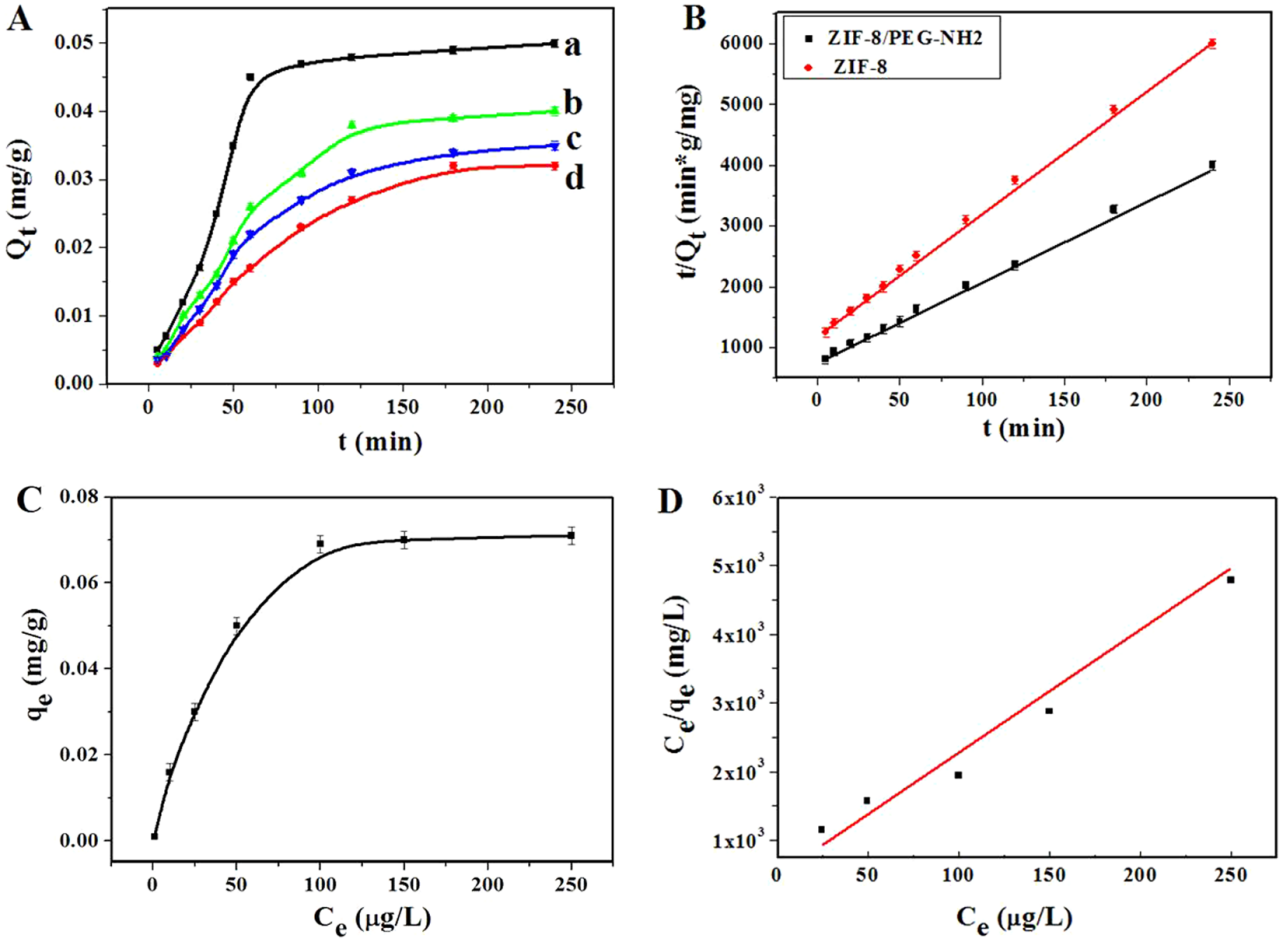
(**A**) Adsorption amount of 4-nonylphenol versus contact time in aqueous solution by using (a) ZIF-8/PEG-NH_2_ membrane, (b) ZIF-8 membrane and (c) PDMS/DVB coating; adsorption amount of bisphenol A versus contact time in aqueous solution by using (d) ZIF-8/PEG-NH_2_ membrane. (**B**) The pseudo-second-order kinetic plot for the adsorption (analyte concentration: 50 μg/L). (**C**) Adsorption isotherm curve of 4-nonylphenol along with (**D**) the linear regression by fitting the equilibrium adsorption data with the Langmuir adsorption model for 4-nonylphenol.

As shown in Fig. 7A, the adsorption of 4-nonylphenol on ZIF-8/PEG-NH_2_ membrane completed in about 60 min, and the adsorption amount was up to 0.05 mg/g. The adsorption process of bisphenol A took 3 h to reach equilibrium, and the adsorption amount was only 0.03 mg/g. The different adsorption results can be explained by the molecular structure of endocrine disruptors. The linear alkyl group with smaller width (3.1 Å) in 4-nonylphenol was accessible to the pore aperture (3.4 Å) of ZIF-8. While, the two benzene rings of bisphenol A had a higher steric hindrance, which made the adsorption capacity lower than 4-nonylphenol. Therefore, 4-nonylphenol was selected to study the isothermal and kinetic properties of the ZIF-8/PEG-NH_2_ because of the high adsorption performance.

Temperature is an important parameter affecting adsorption capacity. At higher temperature the mass transfer rate of analytes is higher, benefiting the adsorption of 4-nonylphenol. However, at the same time, the partition coefficient reduces, because adsorption is an exothermic process, thus the adsorption capacity tends to decreasing. As shown in Supplementary Figure S4, the adsorption capacity increases as the solution temperature is enhanced (up to 50 °C). However, a significant decrease in adsorption capacity is observed when the temperature increases up to 60 °C. According to these results, 50 °C is adopted for subsequent experiments.

We compared the adsorption performance of ZIF-8/PEG-NH_2_ membrane obtained at 20 °C, 40 °C, 60 °C and 80 °C, respectively. The adsorption performance was improved with the reaction temperature increasing to 60 °C (Supplementary Figure S5). However, when the temperature increased to 80 °C, adsorption performance became bad slightly. So, the opportune temperature was 60 °C.

The adsorption behavior of 4-nonylphenol on commercial PDMS/DVB coating and ZIF-8 membrane was investigated under the same adsorption condition. The obtained results are shown in Fig. 7A. Both ZIF-8 and PDMS/DVB showed poor adsorption ability for 4-nonylphenol and an obvious improvement was observed for the prepared ZIF-8/PEG-NH_2_, which was resulted from the synergetic effect of ZIF-8 and PEG-NH_2_. To our knowledge, PEG-NH_2_ is also a good adsorbent, it has good adsorption capacity for 4-nonylphenol.

The adsorption data of 4-nonylphenol were fitted with the pseudo-second order kinetic model using eqn (1),

1 $$\frac{t}{{Q}_{t}}=\frac{t}{{Q}_{e}}+\frac{1}{{K}_{2}{{Q}_{e}}^{2}}$$

where K_2_ is the rate constant of the pseudo-second-order adsorption (g/mg/min), Q_t_ is the amount of analyte adsorbed at time t (mg/g), and Q_e_ is the amount of analyte adsorbed per mass unit of adsorbent at equilibrium (mg/g). As can be seen in Fig. 7B, for ZIF-8/PEG-NH_2_ membrane, a good linear relationship is obtained between time (t) and t/Q_t_ with a correlation coefficient of 0.9972. And the adsorption rate constant (K_2_) is determined to be 2.4 × 10^−3^ g/mg/min, which is higher than that of ZIF-8 in this work (1.0 × 10^−3^ g/mg/min). Moreover, the calculated Q_e_ value agrees with the experimental data, suggesting a pseudo-second-order kinetic model for the adsorption process of 4-nonylphenol on the as-prepared ZIF-8/PEG-NH_2_ membrane.

### Adsorption isotherms for 4-nonylphenol

The adsorption capacity, defined as the maximum amount of 4-nonylphenol adsorbed by a certain amount of adsorbent, is an important factor for the evaluation of the as-synthesized ZIF-8/PEG-NH_2_ membrane. In order to determine the adsorption capacity, the adsorption isotherm of 4-nonylphenol on the ZIF-8/PEG-NH_2_ membrane was investigated and the results are shown in Fig. 7C. Remarkably, with the increase of the initial concentration of 4-nonylphenol, the adsorption amount of 4-nonylphenol increased and then reached a plateau, indicating the saturated adsorption of 4-nonylphenol onto ZIF-8/PEG-NH_2_ membrane. The adsorption capacity of 4-nonylphenol on the ZIF-8/PEG-NH_2_ membrane was calculated to be 0.07 mg/g.

The Langmuir adsorption model was employed for describing the adsorption isotherm. By fitting the equilibrium adsorption data with the Langmuir adsorption model, the adsorption capacity of the ZIF-8/PEG-NH_2_ membrane was calculated by eqn (2):

2 $$\frac{{C}_{e}}{{q}_{e}}=\frac{{C}_{e}}{{q}_{\max }}+\frac{1}{{q}_{\max }{K}_{L}}$$

where C_e_ is the equilibrium concentration of 4-nonylphenol in the solution (μg/L), q_e_ is the amount of 4-nonylphenol adsorbed on the adsorbent at equilibrium (mg/g), q_m_ is the mono-layer adsorption capacity (mg/g), and K_L_ is the Langmuir constant (L/μg). The linear regression between C_e_/q_e_ and C_e_ is fitted as shown in Fig. 7D and the correlation coefficient of the line reaches up to 0.9951, indicating that the adsorption of 4-nonylphenol on the ZIF-8/PEG-NH_2_ membrane fits Langmuir’s adsorption model well.

The developed ZIF-8/PEG-NH_2_ membrane was applied for the detection of 4-nonylphenol in river water samples. The detected concentration was ca. 0.21 μg/L.

### Stability and recycling of adsorbents

**Figure 8:**
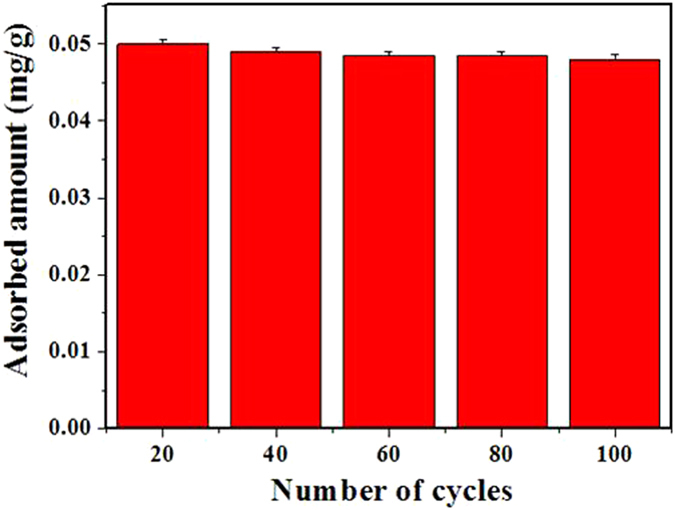
Variation of 4-nonylphenol adsorption on recycled ZIF-8/PEG-NH_2_.

One hundred consecutive adsorption–desorption cycles of the ZIF-8/PEG-NH_2_ membrane was monitored, and the results were shown in Fig. 8. No obvious variation was observed in the adsorption capacity after 100 times reuse. Meanwhile, XRD patterns and N_2_ adsorption–desorption isotherms of ZIF-8/PEG-NH_2_ after 100 adsorption/desorption cycles were investigated. As shown in Supplementary Figure S6a and b, the XRD patterns of ZIF-8/PEG-NH_2_ after 100 adsorption/desorption cycles matched well with the as-synthesized samples, suggesting its excellent stability. Moreover, the results of N_2_ adsorption–desorption isotherms of the reused ZIF-8/PEG-NH_2_ showed that the material maintained a high surface area (1027 m^2^/g). All these results reveal that the structure of ZIF-8/PEG-NH_2_ membrane is stable under the experimental conditions.

## Conclusions

In this paper, a porous PEG-NH_2_ functionalized ZIF-8 membrane was successfully synthesized by a facile coordinate based PSM strategy in methanol solution. Our experiments demonstrated that the reaction time and temperature were crucial for good control of the ZIF-8/PEG-NH_2_ membrane. The membrane presented a fast adsorption rate and high adsorption capacity (0.07 mg/g) for 4-nonylphenol. It was successfully applied for the detection of 4-nonylphenol in environmental water samples. Besides, the prepared ZIF-8/PEG-NH_2_ membrane possessed good stability and reusability. The proposed coordinate based PSM strategy offers an efficient and simple strategy for the preparation of functionalized MOFs membrane with low cost and good performance in water treatment.

## Experimental Section

### Reagents and chemicals

The pencil bars (6.0 cm × 0.3 mm O.D.), purchased from Sinopharm Chemical Reagent Company (China), were cut into length of 2 cm and cleaned (see the Supporting Information). Zinc chloride (≥98%) and sodium formate dihydrate (≥99.5%) were supplied by Sinopharm Chemical Reagent Company. 2-Methylimidazole (Hmim, 99%), methanol anhydrous (≥99.5%), 1-(3-aminopropyl)-3-methylimidazolium bromide ([APMIM]Br, 99%), alpha monomethoxy-omega-amino poly(ethylenglycol) (PEG-NH_2_, 5000 MW), ethylenediamine (EDA, 99%), 4-nonylphenol (99%) and bisphenol A (99%) were purchased from Sigma-Aldrich Chemical. Co. Ltd. All chemicals were used as received from the suppliers without further purifications. The stock solutions of phenolic endocrine disruptors at 1 mg/mL were prepared using methanol as the solvent, and stored at 4 °C. Working solutions with specific concentrations were prepared by step-by-step dilution with methanol just before use. Ultrapure water was used throughout this work.

### IL functionalization of the pencil bar surface

[APMIM]Br (0.2 mL) was dispersed in 1 mL DMF in a 2 mL glass tube. Then the treated pencil bar was immersed in IL solution at 25 °C for 0.5 h, followed by placed in an oven at 80 °C for 6 h. The process was repeated for 3 cycles to ensure complete and uniform coverage. After the coating process, the pencil bar was placed in a desiccator at room temperature for 12 h.

### Synthesis of ZIF-8 membranes on IL-modified pencil bar surface

The ZIF-8 layer was prepared according to the procedure reported elsewhere with minor modifications^48^. Zinc chloride (0.3 g) was dissolved in 10 mL methanol (solution A). IL-treated or IL-free pencil bars were immersed in this solution for 5 min to fix Zn^2+^. 2-Methylimidazole (0.66 g) was dissolved in 10 mL methanol (solution B). Then two solutions were mixed (solution C) and transferred to a Tefion-lined autoclave, and the pencil bars were vertically placed into the autoclave to allow crystal growth at 110 °C for 18 h. After cooling to room temperature, the ZIF-8 coated pencil bars were washed with methanol and dried at room temperature. The ZIF-8 coated pencil bars were activated under N_2_ atmosphere at 250 °C for 10 h and preserved in a vacuum desiccator.

### Fabrication of ZIF-8/PEG-NH_2_ membrane by coordination-based PSM strategy

PEG-NH_2_ (0.02 g) was dissolved in 2 mL methanol solution. The ZIF-8 coated pencil bar was immersed in the solution, and kept in a pre-heated oven (60 °C) for 3 h. After that, the pencil bar was washed two times with methanol to remove non-grafted PEG-NH_2_ and dried overnight at 50 °C in a vacuum.

For comparing study, ZIF-8/methoxy polyethylene glycol (ZIF-8/mPEG) and ZIF-8/EDA were synthesized by the same procedure as mentioned above except by immersing ZIF-8 coated pencil bar in mPEG methanol solution (10 mg/mL) or EDA aqueous solution (20%, V/V).

### Fabrication of ZIF-8/PEG-NH_2_ membrane by one-pot (denoted as One-pot)

Modulated synthesis was employed^49^ for the preparation of One-pot. PEG-NH_2_ (0.1 g) was added into solution C. The subsequent procedure is similar to that described for ZIF-8 membrane.

### Adsorption experiments

The adsorption behaviors of two phenolic endocrine disruptors on ZIF-8/PEG-NH_2_ membrane were studied at a controlled temperature. The ZIF-8/PEG-NH_2_ coated pencil bar was assembled into a homemade solid phase microextraction (SPME) device (described in Ref. 50) and then conditioned in the gas chromatography (GC) injector at 300 °C under nitrogen for 1 h prior to use. To initiate the experiments, 10 mL aqueous solution containing phenols (50 μg/L) was adjusted to pH 3 with 0.1 M HCl and placed in a 15 mL glass vial capped with polytetrafluoroethylene coated septum and the ZIF-8/PEG-NH_2_ coated pencil bar was exposed to the headspace of phenolic aqueous solution. The solution was well stirred at 50 °C for a fixed time (60 min). After adsorption, the pencil bar was withdrawn back to the needle protective shells, then the needle was transferred to the GC injection port for thermal desorption at 300 °C for 5 min, the adsorption amount was measured with a FID detection. For a kinetic study, the adsorption was carried out for predetermined time intervals (i.e. 5, 10, 20, 40, 60, 90, 180, and 240 min) to investigate the effect of contacting time on adsorption. The isothermal adsorption for ZIF-8/PEG-NH_2_ was measured by varying the initial concentrations of endocrine disruptors (from 1 to 250 μg/L).

The adsorption of 4-nonylphenol from environmental water samples was described in the Supporting Information.

### Characterization

The scanning electron microscopy (SEM) images were obtained using an LEO 1530 field emission SEM (Carl Zeiss NTS GmbH, Germany). The component analysis of the polymers was performed with an energy dispersive spectroscopy (EDS). X-ray diffraction data (XRD) were recorded with a Bruke D8 diffractometer (Germany) using Cu Kα radiation (40 kV, 40 mA) with a Ni filter in the range of 5–55°. FT-IR spectra were recorded with a Nexus-670 Fourier transform infrared spectrometer (Nicolet, USA). Prior to FT-IR analysis, the samples were dried under vacuum at 95 °C for 24 h, and analytical grade KBr was dried for 24 h in an oven at 120 °C. The sample and KBr were finely ground using an agate mortar and pestle, then pressed into a pellet for analysis. Nitrogen adsorption−desorption measurements were conducted with a Micromeritics ASAP-2020 M analyzer at 77 K. The sample was degassed at 150 °C for 6 h in vacuum before the tests. The Brunauer, Emmet, and Teller (BET) method was used to calculate the surface area of the adsorbents. Thermogravimetric analysis (TGA) of ZIF-8 was performed on a Netzsch-209 thermal gravimetric analyzer (Bavaria, Germany) from room temperature to 700 °C in flowing N_2_ at heating rate of 10 °C/min.

## Electronic supplementary material

Supplementary Information
