# Supplementary material for: A Poly(ethylenglycol) Functionalized ZIF-8 Membrane Prepared by Coordination-Based Post-Synthetic Strategy for the Enhanced Adsorption of Phenolic Endocrine Disruptors from Water
Source: Sci Rep. 2017 Aug 21;7:8912. doi: 10.1038/s41598-017-09364-1 (PMC5566371; doi:10.1038/s41598-017-09364-1)
Supplement: Supplementary file 1 — Supplementary Information [file 41598_2017_9364_MOESM1_ESM.doc]

**Supporting information for**

**A** **Poly(ethylenglycol) Functionalized ZIF-8 Membrane Prepared by Coordination-Based Post-Synthetic Strategy for the Enhanced Adsorption of Phenolic Endocrine Disruptors from Water**

Mian Wu, Xiafei Guo, Faqiong Zhao, Baizhao Zeng[[1]](#footnote-2)*

Key Laboratory of Analytical Chemistry for Biology and Medicine (Ministry of Education), College of Chemistry and Molecular Sciences, Wuhan University, Wuhan 430072, Hubei Province, P. R. China

Corresponding author: Baizhao Zeng

Tel. : 86-27-68752701

Fax : 86-27-68754067

**Supplement of experimental section**

**The treatment of pencil bar**

The pencil bar was dipped into a 1.0 M NaOH solution (70 °C) for 30 min to enhance surface roughness. Subsequently, the pretreated pencil bar was washed repeatedly with distilled water and ethanol with the aid of sonication, followed by drying in an oven at 100 °C for 5 h.

**Instrumentation**

The heating and stirring of the samples were carried out by using a model S10-3 heater-magnetic stirrer (Shanghai, China). The analysis of phenolic endocrine disruptors was performed on a GC-2010 gas chromatography system (Shimadzu Corporation, Japan) fitted with a split/splitless injection chamber, HP-5 column (30 m, 0.25 mm i.d., 0.25 μm film thickness) (USA) and a flame ionization detector (FID). A GC solution chromatographic workstation program (Shimadzu Corporation) was used to process chromatographic data. Ultrapure nitrogen was used as carrier gas at a constant flow rate of 1 mL/min. Hydrogen and air ﬂow rates were maintained at 40 mL/min and 400 mL/min, respectively. The temperatures of capillary splitless injector and detector were 300 °C. The column temperature was initially set at 50 °C for 3 min, ramped at 10 °C/min to 140 °C, ramped at 5 °C/min to 200 °C, ramped at 10 °C/min to 220 °C, and held for 4 min, and the total run time was about 30 min. The SPME device was laboratory-made. Commercial 65 μm polydimethylsiloxane/divinylbenzene (PDMS/DVB) SPME ﬁber was purchased from Supelco (Bellefonte, PA).

**Adsorption of 4-nonylphenol from environmental water samples**

Polluted river water samples (Wuhan, China) were collected, and centrifuged at 5000 rpm for 2 min. The supernatants were collected in precleaned glass bottles. Then 10 mL river water sample was adjusted to pH 3 with 0.1 M HCl and placed in a 15 mL glass vial capped with polytetrafluoroethylene coated septum and the ZIF-8/PEG-NH_2_ coated pencil bar was exposed to the headspace of phenolic aqueous solution. The solution was well stirred at 50 °C for 50 min. After adsorption, the pencil bar was withdrawn back to the needle protective shells, then the needle was transferred to the GC injection port for thermal desorption at 300 °C for 5 min.

**Supporting figures**


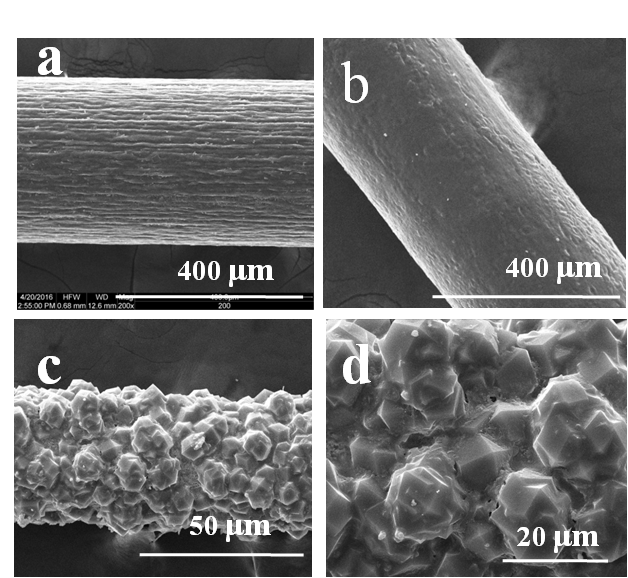


**Figure S1** SEM images of (a) treated pencil bar, (b) IL-modified pencil bar, (c and d) ZIF-8 membrane grown on IL-free pencil bar. (c) low magnification, (d) high magnification.


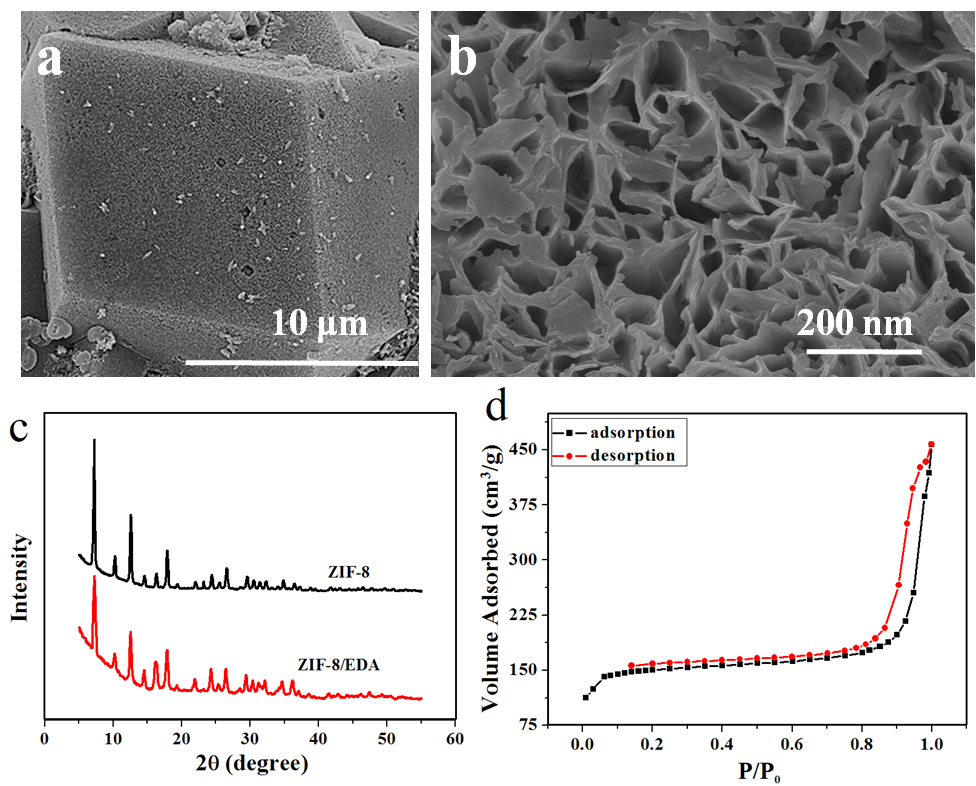


**Figure S2** SEM images (a and b), XRD patterns (c) and N_2_ adsorption–desorption isotherms (d) of the prepared ZIF-8/EDA composites. (a) low magnification, (b) high magnification.


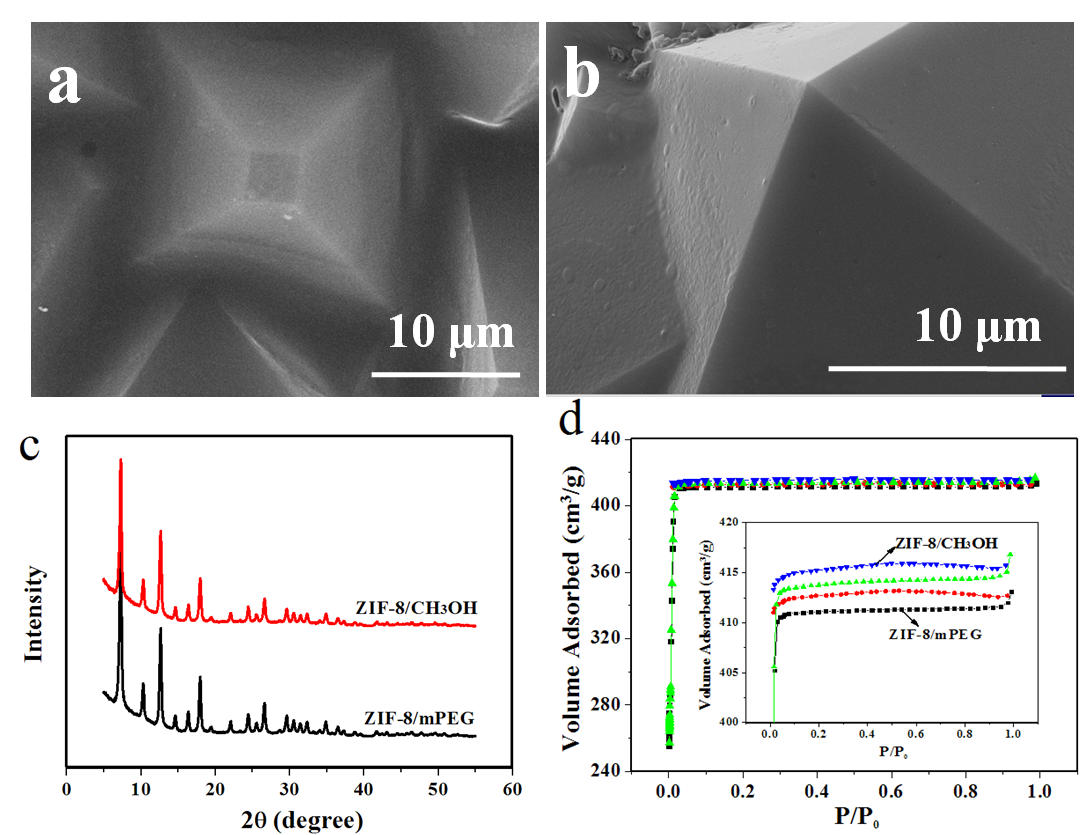


**Figure S3** SEM images of ZIF-8/CH_3_OH (a) and ZIF-8/mPEG (b), XRD patterns (c) and N_2_ adsorption–desorption isotherms (d) of the prepared ZIF-8/CH_3_OH and ZIF-8/mPEG composites, respectively.


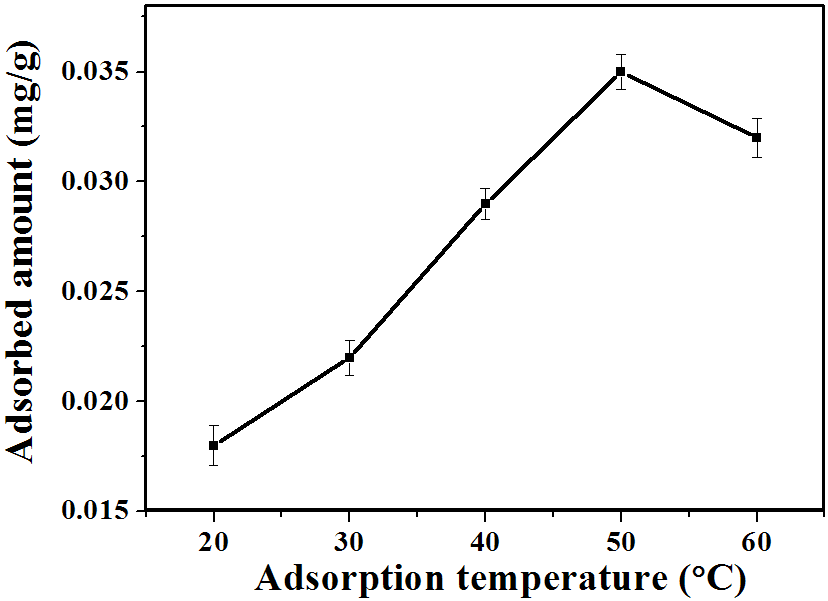


**Figure S4** Effect of adsorption temperature on adsorption efficiency of ZIF-8/PEG-NH_2_ membrane for 4-nonylphenol. Concentrations of 4-nonylphenol, 50 μg/L; adsorption time, 50 min. Error bars show the standard deviation (n = 3).


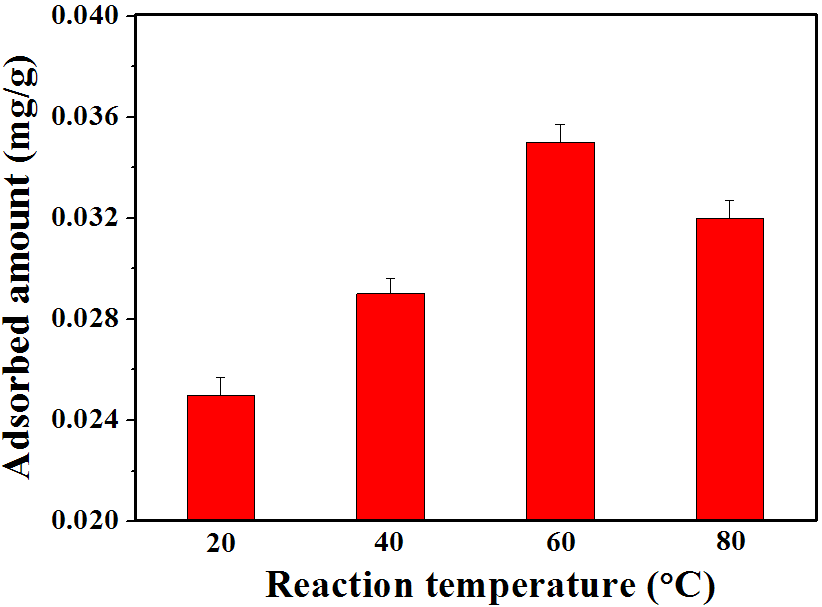


**Figure S5** Comparison of the adsorption performance of ZIF-8/PEG-NH_2_ membrane obtained at 20 °C, 40 °C, 60 °C and 80 °C, respectively. Concentrations of 4-nonylphenol, 50 μg/L; adsorption temperature, 50 °C; adsorption time, 50 min. Error bars show the standard deviation (n = 3).


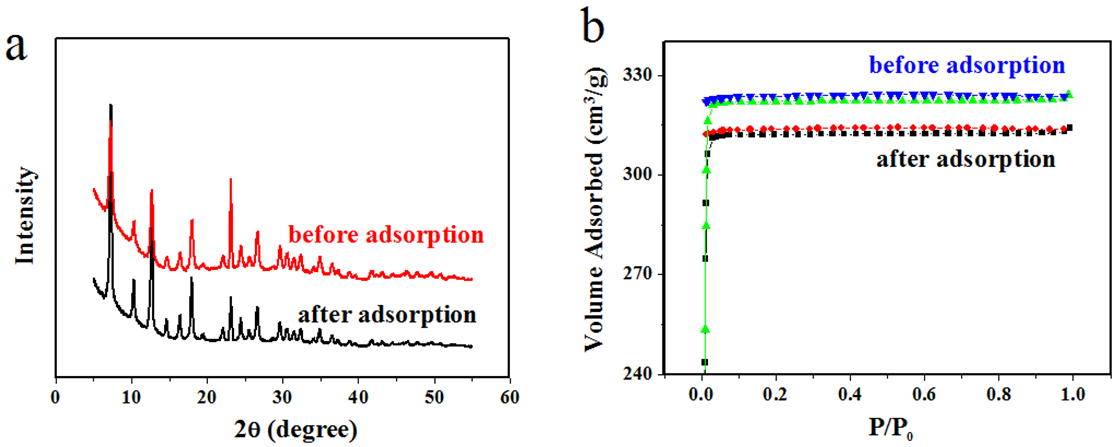


**Figure S6** XRD patterns (a) and N_2_ adsorption-desorption isotherms (b) of ZIF-8/PEG-NH_2_ membrane after 100 adsorption/desorption cycles of 4-nonylphenol.

1. ^^ Corresponding author. Tel: 86-27-68752701, Fax: 86-27-68754067.

   E-mail address: bzzeng@whu.edu.cn [↑](#footnote-ref-2)
